# Impact of language on functional connectivity for audiovisual speech integration

**DOI:** 10.1038/srep31388

**Published:** 2016-08-11

**Authors:** Jun Shinozaki, Nobuo Hiroe, Masa-aki Sato, Takashi Nagamine, Kaoru Sekiyama

**Affiliations:** 1 Department of Systems Neuroscience, School of Medicine, Sapporo Medical University, Sapporo, Japan; 2 ATR Neural Information Analysis Laboratories, Seika-cho, Japan; 3 Division of Cognitive Psychology, Faculty of Letters, Kumamoto University, Kumamoto, Japan

## Abstract

Visual information about lip and facial movements plays a role in audiovisual (AV) speech perception. Although this has been widely confirmed, previous behavioural studies have shown interlanguage differences, that is, native Japanese speakers do not integrate auditory and visual speech as closely as native English speakers. To elucidate the neural basis of such interlanguage differences, 22 native English speakers and 24 native Japanese speakers were examined in behavioural or functional Magnetic Resonance Imaging (fMRI) experiments while mono-syllabic speech was presented under AV, auditory-only, or visual-only conditions for speech identification. Behavioural results indicated that the English speakers identified visual speech more quickly than the Japanese speakers, and that the temporal facilitation effect of congruent visual speech was significant in the English speakers but not in the Japanese speakers. Using fMRI data, we examined the functional connectivity among brain regions important for auditory-visual interplay. The results indicated that the English speakers had significantly stronger connectivity between the visual motion area MT and the Heschl’s gyrus compared with the Japanese speakers, which may subserve lower-level visual influences on speech perception in English speakers in a multisensory environment. These results suggested that linguistic experience strongly affects neural connectivity involved in AV speech integration.

Visual information about lip and facial movements plays a large role in vocal speech perception. This has been shown to have an enhancing effect for audiovisual (AV) congruent speech (e.g., Sumby, 1954)^1^, and a disrupting effect for AV incongruent speech, such as in the McGurk illusion^2^. This enhancement includes not only increased accuracy in noisy circumstances^1^, but also increased speed in perceiving congruent AV speech compared with auditory-only (AO) speech in quiet circumstances^3^,^4^. Such a temporal facilitation is thought to be due to orofacial movements starting slightly before the auditory onset in natural speech production^3^,^5^. This time lag may allow the brain to anticipate auditory signals based on visual information^3^,^4^,^5^. On the other hand, incongruent AV speech often induces the McGurk illusion, in which the percept is different from that for AO speech, for example, a combination of the auditory /ba/ and the visual /ga/ may be perceived as /da/2,6.

Both the enhancing and disrupting effects of AV speech have contributed to the documentation of the multisensory nature of speech perception, that is, how closely auditory and visual speech are processed together. However, several previous studies have found that this close coupling may not be universal, for example, native speakers of Japanese show a much weaker McGurk effect than those of English^7^,^8^,^9^,^10^,^11^. One characteristic of Japanese speakers experiencing AV incongruent speech stimuli is that they rely on auditory speech and perceive mouth movements as “incongruent with the real speech”. This is in contrast with English speakers who easily integrate auditory and visual speech and do not notice the incongruity^7^.

It has also been shown that it is between ages 6 and 8 years old when these interlanguage differences between English and Japanese become developmentally apparent in AV speech perception^10^. Although pre-lingual infants recognize voice-mouth matching for vowels^12^,^13^ and may show some early signs for the occurrence of the McGurk effect^14^,^15^ (also see Desjardins (2004)^16^), preschool and school-age young children still tend to rely on auditory speech more so than adults for the McGurk-type incongruent AV speech^2^,^10^,^17^,^18^,^19^. Thus, young children still require time to achieve AV speech integration to attain the level of adult native English speakers. This is presumably related to the fact that lipreading is very difficult for young children^10^,^17^,^18^. Therefore, returning to the cross-linguistic developmental study by Sekiyama and Burnham^10^, the 6-year-olds’ lipreading abilities may have not been high enough to have had an effect on auditory processing, which would have yielded only a weak McGurk effect irrespective of their language background. However, it was striking that Japanese adults remained at a level similar to 6-year-olds in integrating auditory and visual speech, in spite of their increased lipreading ability^10^. It may be that the Japanese language has some characteristics that do not promote the use of visual articulatory information. In consonants, English has 6 visemes^20^,^21^ while Japanese has 3 visemes^22^. A viseme, an analogy of phoneme, is a category within which perceivers cannot further categorize speech sounds due to visual similarity for lipreading. Defining number of visemes as informative, Japanese has a smaller number of phonemes and less informative visual speech^20^,^21^,^22^. Due to such factors, the development of neural connectivity among different brain regions for AV speech perception may be quite different between native speakers of Japanese and those of English. This study investigated these interlanguage differences in neural connectivity.

Previous functional neuroimaging studies on AV integration have shown that the left Superior Temporal Sulcus (STS) is persistently activated for AV integration of speech under various experimental settings^6^,^23^,^24^,^25^,^26^,^27^,^28^,^29^. This is reasonable because the STS is one of the major “higher-order” multisensory convergence zones (Driver (2008) for review^30^). Previous studies in nonhuman primates have shown that the STS receives input from both the auditory cortex and visual cortex^31^,^32^. Nath and Beauchamp (2011) have shown that noisy visual stimuli decrease the input from the visual cortex to the STS, while noisy auditory stimuli decrease the input from the auditory cortex to the STS in audiovisual speech perception^33^. These studies suggested that the STS receives input from both the auditory cortex and visual cortex in humans. It seems that the auditory input via the auditory association cortex and the visual input via the middle temporal visual area (MT) may converge in the STS for perceiving AV integrated speech.

On the other hand, there is increasing evidence for an early influence of visual input on the auditory cortex in multisensory processing, perhaps not mediated by the higher-order multisensory convergence zone (Ghazanfar (2006), Driver (2008), Schroeder (2008) for review^30^,^34^,^35^). A direct anatomical route from visual cortex to auditory cortex has been reported in non-human primates^36^,^37^,^38^,^39^,^40^. In human intracranial electrophysiological study, mouth movement in the AV stimuli activate auditory cortex, 10 ms after the activation of MT^41^, supporting an early influence of visual input on the auditory cortex. A few recent neuroimaging studies have proposed a dual-route model of AV speech perception; in addition to the convergence of afferent sensory inputs in the STS, there is a more direct pathway that allows quick visual influence on auditory speech processing^29^,^42^.

To date, only one neuroimaging study has tested native speakers of Japanese for speech perception by facial and vocal stimuli^25^. The results suggested that the Japanese had little multisensory integration for AV incongruent (McGurk-type) speech presented under a relatively high auditory signal-to-noise ratio. On the other hand, they did integrate AV speech when the auditory signal-to-noise ratio was lower, with substantial occurrence of the McGurk effect and left STS activation.

In order to compare native speakers of Japanese and English, the present study focused on the temporal facilitation effect for AV congruent speech, rather than the McGurk effect for AV incongruent speech. This is because a previous study indicated that neural responses for multisensory integration may be more clearly observed for AV congruent than incongruent speech^3^. On the other hand, focusing on the temporal facilitation effect for AV congruent speech can avoid very noisy conditions for capturing AV integration in Japanese speakers, which is important to make a fair comparison between native speakers of Japanese and English, because interlanguage differences tend to be clearer when auditory speech is intelligible^8^. With the AV congruent speech stimuli, we compared functional connectivity among brain regions between native speakers of Japanese and English. Based on the previous behavioural findings, we predicted a smaller temporal facilitation effect of congruent visual speech, as well as less/weaker brain functional connectivity between auditory and visual regions for native speakers of Japanese than those of English.

## Results

### Behavioural experiment

The task of the participant was to decide what he/she perceived by choosing from “ba”, “da”, and “ga”, and pressing one of three buttons with the left hand as accurately and quickly as possible. There were three conditions (AV, AO, and visual only (VO)).

**Figure 1:**
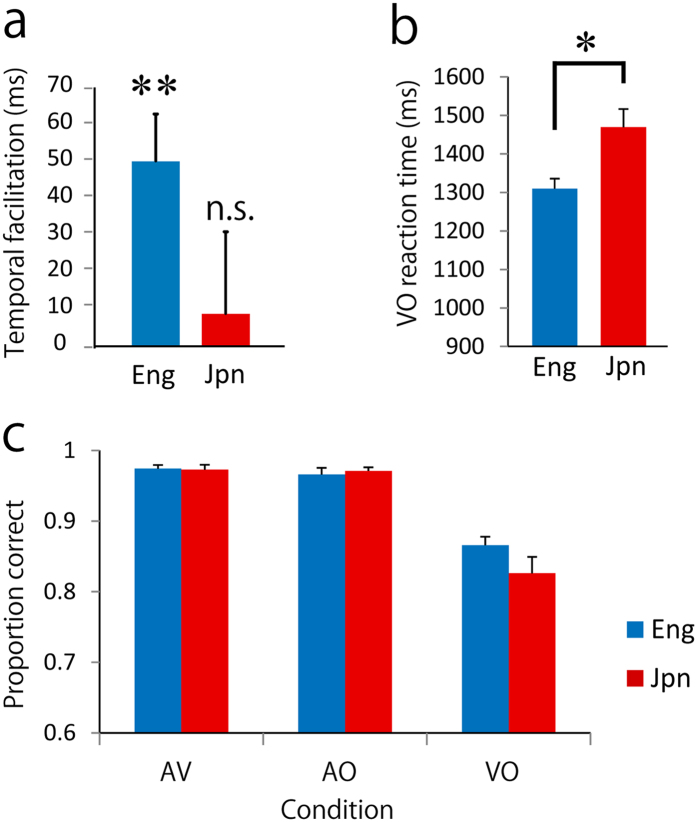
Behavioural results. The temporal facilitation of visual speech (shorter RTs for the AV than the AO condition) was found only in English speakers (**a**). Lipreading was significantly faster in English speakers than in Japanese speakers (**b**). The proportion of correct responses was relatively high in all conditions, but those in the VO condition were less than those in the other two conditions (**c**). Eng; native English speakers. Jpn; native Japanese speakers. Error bars mean standard errors. **p < 0.01, *p < 0.05, n.s.; not significant.

To investigate the degree of audiovisual integration, we defined temporal facilitation by visual speech by subtracting the RTs of AV from AO in each group (pooled talker’s effect). The temporal facilitation was 50 ± 13 ms (mean ± standard error) for the English speakers. A one-sample *t*-test showed a significant temporal facilitation compared with zero (*t*_19_ = 3.907, *p* = 0.001, Cohen’s *d* = 0.87). In Japanese speakers, temporal facilitation was 9 ± 22 ms, and a one-sample *t*-test did not show a significant temporal facilitation (*t*_21_ = 0.396, *p* = 0.696, *d* = 0.08) (Fig. 1a).

We tested whether lipreading was faster in English speakers than in Japanese speakers. A two-sample t-test (pooled talker’s effect) showed that lipreading was significantly faster in English speakers than in Japanese speakers (*t*_40_ = 2.894, *p* = 0.006, *d* = 0.89) (Fig. 1b).

To summarize, the temporal facilitation effect of congruent visual speech (i.e., AV condition) was significant only in English speakers, but not in Japanese speakers, and English speakers were much quicker than Japanese speakers at lipreading (VO condition) (by 160 ms on average).

The accuracy was high in both groups. In English speakers, the accuracies and standard errors were 97.4 ± 0.5%, 96.6 ± 0.9%, and 86.6 ± 1.2% in the AV, AO, and VO conditions, respectively. In Japanese speakers, the accuracies and standard errors were 97.2 ± 0.7%, 97.1 ± 0.4%, and 82.6 ± 2.3% in the AV, AO, and VO conditions, respectively (Fig. 1c).

Additional analyses were conducted to investigate subgroup differences (Caucasian versus Asian) in English speakers. The RTs were essentially the same between Caucasian English-speakers and Asian English-speakers (see Supplementary Information). We also compared the behavioural data collected inside and outside the scanner (i.e., between the fMRI and behavioural experiments). The RTs did not significantly differ between behavioural experiments and fMRI experiments (see Supplementary Information).

### fMRI experiment

#### Multisensory and unisensory responses

**Figure 2:**
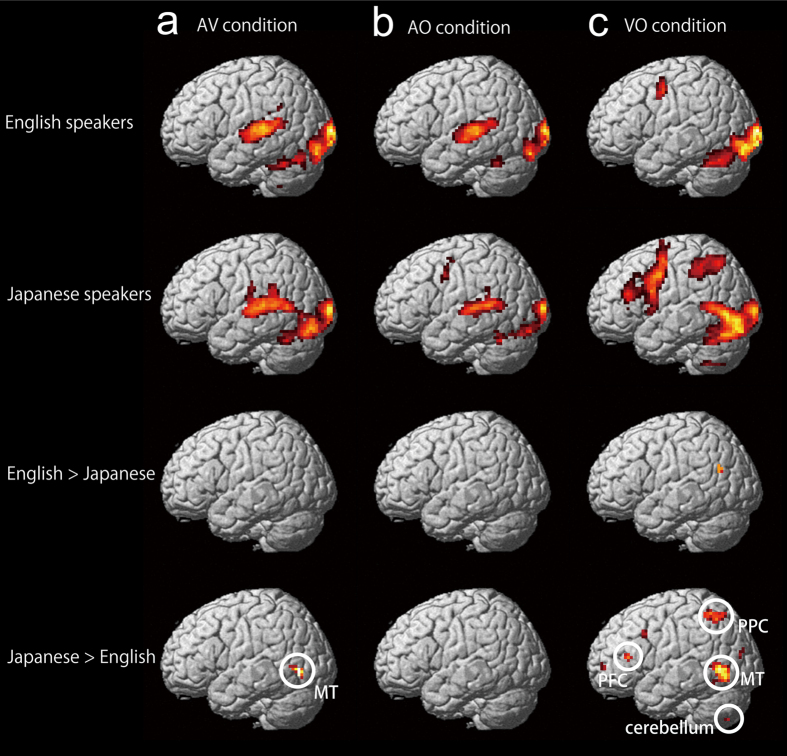
Brain areas activated under AV, AO, and VO conditions. Brain areas activated under the AV condition in native English speakers and native Japanese speakers (voxel level *p* < 0.001, uncorrected; cluster level *p* < 0.05, corrected), and those showing greater activation in native English speakers than Japanese speakers, and those vice versa (**a**) (voxel level *p* < 0.001, uncorrected). The left inferior temporal gyrus, including MT, showed greater activity in native Japanese speakers than in native English speakers. Note that activity in the right hemisphere or medial region is not shown here; instead, they are shown in Table 1. Brain areas activated by AO stimuli in native English speakers and native Japanese speakers (voxel level *p* < 0.001, uncorrected; cluster level *p* < 0.05, corrected), and those showing greater activation in native English speakers than Japanese speakers, and those vice versa (**b**) (voxel level *p* < 0.001, uncorrected). There was no significant difference between groups in activity in the lateral left hemisphere. Note that activity in the right hemisphere or medial region is not shown here; instead, they are listed in Table 1. Brain areas activated by VO stimuli in native English speakers and native Japanese speakers (voxel level *p* < 0.001, uncorrected; cluster level *p* < 0.05, corrected), and those showing greater activity in native English speakers than in native Japanese speakers, and those vice versa (**c**) (voxel level *p* < 0.001, uncorrected). There was no significantly greater activity in the lateral left hemisphere in native English speakers than in native Japanese speakers, except in the left posterior superior temporal gyrus. The left PPC, PFC, and inferior/middle temporal gyrus including MT, as well as cerebellum showed greater activity in native Japanese speakers than in native English speakers. PPC; posterior parietal cortex. PFC; prefrontal cortex.

Stimuli were the same as in the behavioural experiment except only two syllables (/ba/ and /ga/) were used in the fMRI experiment. Figure 2a and Table 1 show areas activated under the AV condition in native English and Japanese speakers. The AV condition involved the bilateral superior temporal gyri, and the occipital cortex including the fusiform gyrus (Fusiform Face Area (FFA)^43^) in both native English and Japanese speakers, while activity in the MT was found only in Japanese speakers. Neural activity in the right precentral gyrus (primary motor cortex (M1)) and medial frontal gyrus (supplementary motor area (SMA)) was also observed, perhaps due to the manual response (Table 1). Significantly greater activity was observed in the posterior cingulate in native English than in Japanese speakers (Table 1), and in the left inferior temporal gyrus including MT in native Japanese than in English speakers in group comparisons.

Figure 2b and Table 1 show areas activated by AO unistimulation in native English and Japanese speakers. The AO stimuli, which consisted of unisensory audio stimuli with a still face, activated the bilateral superior temporal gyri, the visual area including the FFA, and motor related areas including the right M1 and SMA. In group comparisons, a few regions showed significant group differences (Table 1), but their cluster sizes were relatively small.

VO unistimulation induced neural activity in the visual cortex including FFA, superior/middle temporal gyrus, and premotor cortex in both groups (Fig. 2c, Table 1). Only limited areas showed greater activation for English than Japanese speakers (Table 1), while various regions showed greater activation for Japanese than for English speakers (Fig. 2c): these regions included the bilateral inferior/middle temporal gyrus including MT, posterior parietal cortex (PPC), a few regions in prefrontal cortex (PFC), and cerebellum.

### Functional connectivity

#### AV condition

**Figure 3:**
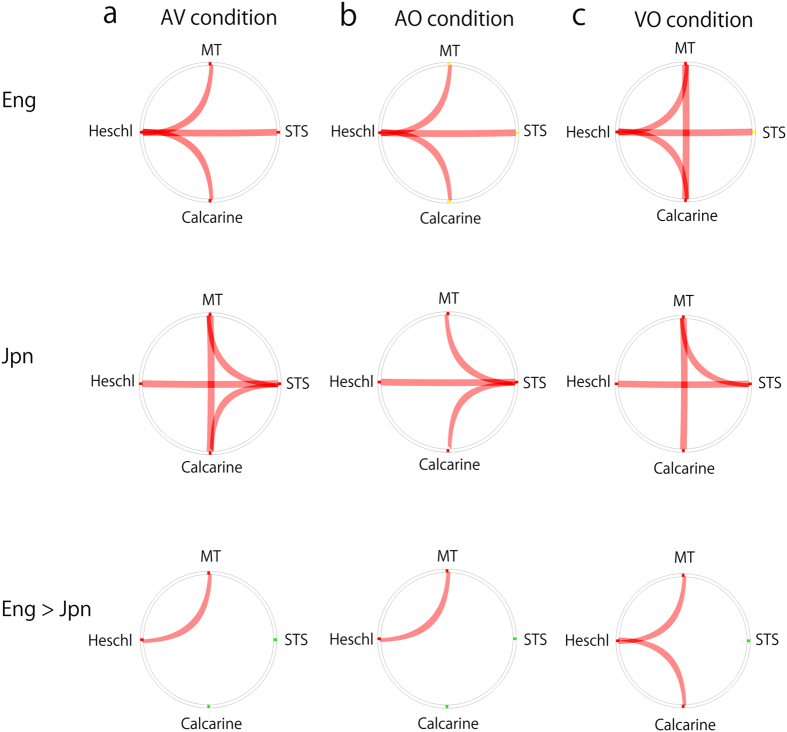
Functional connectivity. Functional connectivity among our 4 ROIs in the left hemisphere in the AV condition (**a**), AO condition (**b**), and VO condition (**c**) (*p* < 0.05 false discovery rate (FDR) corrected). Positive correlation is shown in red, and negative correlation is shown in blue, though there was no negative correlation. In the analysis of group differences, connectivity shown in red means stronger connectivity in English speakers than Japanese speakers (bottom row). Eng; English speakers. Jpn; Japanese speakers.

In English speakers, Heschl-centred connectivities were observed, that is, significant MT-Heschl (*p* < 0.001, *Z* = 0.27 (*Z*: Fisher’s *Z*-transformation of correlation coefficients *r*)), Calcarine-Heschl (*p* = 0.036, *Z* = 0.10), and Heschl-STS (*p* = 0.001, *Z* = 0.17) connectivities. Inconsistent with a model of integration in the STS^29^, the MT-STS connectivity was not significant (*p* = 0.157, *Z* = 0.05). In contrast, Japanese speakers showed STS-centred connectivities (Calcarine-STS (*p* = 0.046, *Z* = 0.09), MT-STS (*p* = 0.006, *Z* = 0.12), and Heschl-STS (*p* < 0.001, *Z* = 0.19)) as well as a visual connectivity (Calcarine-MT (*p* = 0.001, *Z* = 0.14)). The analysis of group differences showed that English speakers had a stronger low-level cortico-cortical connectivity in MT-Heschl than Japanese speakers (*p* < 0.001, *Z* = 0.21) (Fig. 3a).

#### AO condition

In English speakers, the same Heschl-centred connectivities as the AV condition were observed (MT-Heschl (*p* < 0.001, *Z* = 0.24), Calcarine-Heschl (*p* < 0.001, *Z* = 0.13), and Heschl-STS (*p* < 0.001, *Z* = 0.17)). In Japanese speakers, similar to the AV condition, STS-centred connectivities were found (Calcarine-STS (*p* = 0.024, *Z* = 0.07), MT-STS (*p* = 0.034, *Z* = 0.07), and Heschl-STS (*p* < 0.001, *Z* = 0.19)), with a non-significant visual connectivity (Calcarine-MT (*p* = 0.107, *Z* = 0.10)). Consistent with the AV condition, the MT-Heschl connectivity was stronger in English speakers than Japanese speakers (*p* = 0.001, *Z* = 0.19) (Fig. 3b).

#### VO condition

In English speakers, visual connectivity (Calcarine-MT (*p* = 0.043, *Z* = 0.09)) was added to the Heschl-centred connectivities found in AV and AO conditions (MT-Heschl (*p* < 0.001, *Z* = 0.22), Calcarine-Heschl (*p* = 0.001, *Z* = 0.10), and Heschl-STS (*p* = 0.001, *Z* = 0.15)). In Japanese speakers, the pattern of significant connectivities was similar to the AV condition (MT-STS (*p* < 0.001, *Z* = 0.13), and Heschl-STS (*p* < 0.001, *Z* = 0.17)), with non-significant Calcarine-STS connectivity (*p* = 0.161, *Z* = 0.05). The MT-Heschl and Calcarine-Heschl connectivities were stronger in English speakers than Japanese speakers (*p* < 0.001, *Z* = 0.27 and *p* = 0.046, *Z* = 0.10, respectively) (Fig. 3c).

Apart from these connectivities, BOLD responses in these ROIs are shown as the percent signal changes in the AV condition (in Supplementary Information).

## Discussion

This study investigated the neural basis of interlanguage differences between native speakers of English and Japanese in AV speech perception. We predicted a smaller temporal facilitation effect of congruent visual speech, as well as less/weaker brain functional connectivity between auditory and visual regions for native speakers of Japanese than those of English. We used AV congruent stimuli and examined 1) the visual facilitation effect in reaction times as a behavioural measure, and 2) the functional connectivity among the different brain regions. Consistent with a previous study^10^, the behavioural experiment showed a visual facilitation effect on reaction time in native English speakers, but not in native Japanese speakers.

The functional connectivity analysis in the present study indicated that low level connectivity between the visual cortex (Calcarine/MT) and auditory cortex (Heschl) was observed only in English speakers under AV, AO, and VO conditions, suggesting that early visual input to Heschl may occur only for English speakers in audiovisual speech perception. Such low level connectivity may be realized via thalamus, the sub-cortical relay centre for various modalities of signalling^44^,^45^, and may contribute to multisensory processing^46^. Consistent with this view, an additional functional connectivity analysis including Thalamus ROI showed significant Thalamus-Calcarine, Thalamus-Heschl, and Thalamus-MT connectivities in English speakers under AV conditions (FDR corrected *p* < 0.05 (two-tailed)), while in Japanese speakers, such connectivities were not significant (see Supplementary Information (Fig. S2)). Therefore, the low-level areas such as the Heschl and Thalamus may have a larger role in English speakers’ audiovisual interaction, whereas, Japanese speakers may merge visual and auditory information only at the STS, a higher integration site, via cortico-cortical connectivity (Calcarine/MT-STS connectivity and Heschl-STS connectivity). Although significant STS-centred connectivities were found in Japanese speakers, the effect sizes of visual-related connectivities were relatively small^47^ (e.g., *Z* = 0.12 for MT-STS under AV condition), suggesting that visual input to the STS may be weak and STS-centred connectivities in Japanese speakers may be moderately tied.

The STS is a core region for AV integration in humans^6^,^23^,^25^,^26^,^33^,^48^,^49^,^50^,^51^,^52^,^53^,^54^,^55^. Consistent with this view, Japanese speakers showed the STS-centred connectivities, that is, Calcarine/MT-STS connectivity and Heschl-STS connectivity in the present study. Thus, the cortico-cortical network may contribute to audiovisual integration in the STS. However, in English speakers, the functional connectivity analysis did not find significant Calcarine/MT-STS connectivity. Rather, significant Heschl-centred connectivities were observed. This is consistent with the observation of the early influence of visual input on the auditory cortex (from Calcarine/MT to Heschl) in multisensory processing, possibly not mediated by the STS^30^,^34^,^35^. This low-level connectivity may have realized the greater visual temporal facilitation in English speakers we observed. Furthermore, this early AV interplay in the auditory cortex in English speakers is consistent with a previous report on AV interaction in the auditory cortex in native English speakers^42^. In Japanese speakers, we could not observe significant direct connectivity from the visual area to the auditory area, instead, the convergence of auditory and visual inputs seemed to occur only in the STS. This manner of connectivities in Japanese speakers may have caused non-significant temporal facilitation during audiovisual integration.

Consistent with a previous study^25^ showing that AV stimuli activated the left MT in native Japanese speakers, the left MT showed significantly greater activation in Japanese speakers’ visual-related speech perception (AV and VO), compared with English speakers. This left MT activity in Japanese speakers may be related to their large dependence on a relatively higher-level connectivity (MT-STS) in visual speech processing, whereas English speakers’ visual speech processing is distributed to lower-level connectivities (MT-Heschl, Calcarine-Heschl) including Thalamus (Fig. S2). As another possibility, the greater left MT activation in Japanese speakers may be related to their relatively greater difficulty in handling lipreading information. In the behavioural experiment, the English speakers were much quicker than the Japanese speakers in lipreading. The slower (more difficult) lipreading in Japanese participants may be associated with the much more widely spread brain activation, including in the MT, PPC, PFC, and cerebellum, compared with English participants.

One of the possible reasons for the differences in observed functional connectivity between the English and Japanese speakers may be the difference in language characteristics, such as the greater number of phonemes (14 vowels in English versus 5 vowels in Japanese) and more informative visual speech (6 visemes in English versus 3 visemes in Japanese) in English than in Japanese^20^,^21^,^22^. Such language characteristics (more useful visual cues, more ambiguous auditory cues) in everyday life may foster more significant calcarine/MT-Heschl connectivity for efficient AV speech processing in English speakers as they develop into adults. The present study showed significant Calcarine/MT-Heschl connectivity only in English speakers, suggesting that the functional strength of this low level network may be modulated by language characteristics^30^,^34^,^35^.

## Conclusion

We observed that the level of processing at which visual input influences auditory speech processing may differ between native English speakers and native Japanese speakers. Only English speakers showed significant MT-Heschl connectivity, which may be related to the greater temporal facilitation of visual speech compared with Japanese speakers, suggesting that the language environment during development may alter the brain network.

## Methods

### Participants

Native speakers of English (22 young adults) (English-speaker GROUP) and Japanese (24 young adults) (Japanese-speaker GROUP) were recruited from the Kyoto area in Japan through campus advertisement at several universities. Most of the English speakers were Caucasian, and all of Japanese speakers were Japanese. After excluding a few participants with low accuracy (lower than 0.67 in proportion correct) or response bias (no accurate responses for /ga/) in lipreading (two English and two Japanese speakers), the behavioural data were analysed for twenty English speakers (10 males and 10 females, 15 Caucasians and 5 Asians, mean age was 22.4 years, median length of stay in Japan was 6 months) and 22 Japanese speakers (12 males and 10 females, all of them were Japanese, mean age was 23.9 years, without experience of staying abroad for more than 3 months). For the fMRI experiment, 21 native English speakers (11 males and 10 females, 16 Caucasians and 5 Asians, average age was 22.1 years, median length of stay in Japan was 6 months) and 19 native Japanese speakers (10 males and 9 females, all of them were Japanese, average age was 24.0 years, without experience of staying abroad for more than 3 months) were included for the data analysis. All participants were right-handed, had normal hearing, and normal or corrected to normal vision, and few of them were proficient in their second language (Japanese or English). No English speakers could understand the instructions well in Japanese, and vice versa. We instructed in English (Japanese) for English (Japanese) speakers, that is, in a participant’s native language.

### Ethics Statements

The experimental protocol was approved by the ethical committee of Advanced Telecommunications Research Institute International (ATR), and was in accordance with the Declaration of Helsinki. Written informed consent was obtained from each participant.

### Behavioural experiment

#### Stimuli

The speech stimuli of the behavioural experiment were produced from the articulation of /ba/, /da/, and /ga/ by two male talkers, one native English speaker, and one native Japanese speaker. These phonemes in Japanese are similar to those in English, although recorded consonants and vowels were slightly shorter in Japanese. The recorded speech signals were edited by a digital waveform editing software and a movie editing software so that the onset of the auditory speech was 900 ms from the beginning of each movie file. Video signals were digitized at 29.93 frames/s in 640 × 480 pixels, and audio digitized at 44.1 kHz in 16 bits. The intensity of the speech sound was normalized across different articulations. The duration of each movie file was approximately 1700 ms, and the duration of auditory speech was 400 ms on average. Unisensory stimuli were produced based on the above normalized and time-aligned AV stimuli. The AO stimuli were produced by replacing the visual component of the AV stimuli by the still face of that talker. The VO stimuli were produced by deleting the auditory component of the AV stimuli.

#### Procedure

The behavioural experiment was conducted in a quiet room, outside the MRI scanner. The experiment was controlled by the Presentation software (Neurobehavioral Systems) running on a PC. The participant was seated in front of a 19-in LCD monitor at a 50 cm distance. The video signals were presented on the monitor and the audio signals via tube-type earphones. To approximate the MRI scanner noise, auditory band noise (300 to 12000 Hz, similar to machine noise) was added via an audio mixer at a signal-to-noise ratio of 15 dB (speech was 65 dB and noise was 50 dB). This signal-to-noise ratio should have had little effect on auditory speech intelligibility based on a previous study^10^. The task of the participant was to decide what he/she perceived by choosing from “ba”, “da”, and “ga”, and pressing one of three buttons with the left hand as accurately and quickly as possible. In the AV condition, the participants were instructed to respond as soon as possible after listening to the auditory syllable, and not to respond before the sound onset, because several English speakers claimed that they could identify phonemes by observation of the talkers’ mouth movements without listening them. In the AO condition, the instruction was essentially the same as for the AV condition (i.e., to respond as soon as possible after listening to the auditory syllable). In the VO condition, the task required lipreading, because there was no auditory cue. The three conditions (AV, AO, and VO) were blocked, and the AV condition was conducted first. This was followed by half of the participants tested in an AO to VO order, and the other half in the opposite order. In each condition, two blocks of 60 trials (10 repetitions × 3 stimuli × 2 talkers) were conducted. The first block in each condition was regarded as practice and the second block was analysed. Six kinds of AV clips (3 stimuli × 2 talkers) of 1700 ms duration were presented for pseudo-random order. The interval between two successive AV clips was set randomly from 1000 ms to 1400 ms. A fixation cross pattern was presented during this interval.

#### Data analysis

For each condition (AV, AO, and VO), each participant’s proportion correct and mean RT were calculated. Only correct responses were used for RT analyses. To investigate the degree of audiovisual integration, we defined the temporal facilitation by visual speech by subtracting the RTs of AV from AO in each group. Data was pooled across talkers because there was no significant effect of talker (*p* = 0.115) (see Supplementary Information). A one-sample t-test was conducted for the temporal facilitation in each group. For the RTs in the VO condition, we conducted a two-sample *t*-test between English speakers and Japanese speakers.

We did not conduct any statistical analysis of accuracy because there was a ceiling effect due to the simplicity of the task.

### fMRI experiment

#### Stimuli and tasks

Stimuli were the same as in the behavioural experiment except only two syllables (/ba/ and /ga/) were used in the fMRI experiment. The stimuli were presented in a blocked design by alternating three stimulus blocks and one rest block in an AV-AO-VO-rest pattern. Each of the 4 stimuli (/ba/ and /ga/ of the two talkers) were presented twice in each block with a jittered interval between two successive AV clips (2300 ± 1000 ms) in order to increase vigilance. The duration of each block was 32 s on average. One functional session was composed of four AV-AO-VO-rest sequences. In total, three functional sessions were repeated.

The participants’ task was the same as the behavioural experiment, and the participants were asked to report what they perceived by pressing a button (/ba/ or /ga/) with their left hand during fMRI scanning. There were 8 trials within a single 32-second block. Participants were instructed to press a button on each trial (i.e., 8 times within a single block).

#### Procedure

Each participant lay supine on a scanner bed, with a button response device held in the left hand. Sound was delivered via MR-compatible headphones. Auditory stimuli were presented with a sufficiently loud volume compared with the MR scanner noise. We estimated that the SNR might be over 10 dB in the fMRI scanner in the present study because the accuracy in the scanner (98.3% under AO condition in both groups) in the present study was higher than that of a previous study^10^ (>~95% under AO condition in both groups) in which an SNR was over 10 dB. The participants viewed visual stimuli that were back-projected onto a screen through a built-in mirror. Foam pads were used to minimize head motion.

#### Image acquisition

Functional MRI experiments were conducted on a 3-Tesla whole-body scanner equipped with a 12-ch phased array coil (Siemens Tim Trio, Erlangen, Germany). Functional images were obtained in a T2*-weighted gradient-echo echo-planar imaging sequence. The image acquisition parameters were as follows: repetition time (TR) = 3.0 s; echo time (TE) = 30 ms; flip angle (FA) = 80°; field of view (FOV) = 192 mm; matrix = 64 × 64; 50 interleaved axial slices with 3-mm thickness without gaps (3-mm cubic voxels). The first four images were not saved to allow for signal stabilization. For anatomic images, T1-weighted three-dimensional structural images were obtained using a magnetization-prepared rapid-gradient echo sequence.

#### General linear model (GLM) analysis

The fMRI data were analysed with SPM8, using the principles of the GLM^56^. The functional images were corrected for differences in slice-acquisition timing, and were then spatially realigned to the first image of the initial run to adjust for residual head movements. The realigned images were spatially normalized to fit to a Montreal Neurological Institute (MNI) template^57^ based on the standard stereotaxic coordinate system^58^. Subsequently, all images were smoothed with an isotropic Gaussian kernel of 8-mm full-width at half-maximum (FWHM), except for functional connectivity analysis. Each of the three stimulus conditions (AV, AO, VO) and 6 head motion parameters were separately modelled as regressors for the first-level multi-regression analysis. This analysis was performed for each participant to test the correlation between the MRI signals and boxcar functions convolved with the canonical hemodynamic response function. Global signal normalization was performed only between runs. Low-frequency noise was removed using a high-pass filter with a cut-off of 128 s, and serial correlation was adjusted using an AR(1) model. By applying the appropriate linear contrast to the parameter estimates, mean effect images reflecting the magnitude of correlation between the signals and the model of interest were computed. These were used for the subsequent second-level random-effect model analysis. Group-level statistical parametric maps were produced using the one-sample *t*-test. A two-sample *t*-test was calculated to clarify group differences between native English speakers and native Japanese speakers. These results are shown at a height threshold of *p* < 0.001 (uncorrected) with an extent threshold of 10 voxels^59^,^60^,^61^. These activities were overlaid onto MNI template brain.

#### Functional connectivity analysis

Analysis of functional connectivity was performed using the CONN toolbox (www.nitric.org/projects/conn)^62^, by investigating the bivariate correlation of time courses between two ROIs. By using the “CompCor” method^63^, which is able to remove biases related to non-neural sources (such as respiration or cardiac activity), we removed principle components associated with segmented white matter (WM) and cerebrospinal fluid (CSF) for each individual participant. The time courses of the WM and CSF seeds were regressed out. An additional 12 motion regressors (6 realignment parameters and their first derivatives), due to head movement, were regressed out. The effect of each condition was also regressed out, the resulting time course data were orthogonal with task design. This procedure could avoid circularity. The time course data were filtered from 0.008 Hz to 0.1 Hz.

We focused only on the left hemisphere to define ROIs because the left hemisphere is language dominant^33^. Previous studies have shown a significant positive interpersonal correlation between left STS activity and the likelihood of the McGurk effect^6^,^52^. A previous Transcranial Magnetic Stimulation (TMS) study found inhibition of the McGurk effect by left STS TMS^55^. In our study, MT showed stronger activity in Japanese speakers than in English speakers in the AV and VO conditions only in the left hemisphere. Based on these previous findings and our data, we decided to focus only on the left hemisphere to define ROIs. We defined 4 ROIs (left STS, Heschl’s gyrus (Heschl), calcarine sulcus (Calcarine), and middle temporal visual area (MT) as seeds for functional connectivity analysis. These ROIs were defined by conjunction of GLM functional results (group analyses per group, except for STS) and anatomical atlas. The centre coordinate was defined as the peak coordinate of activity in group analysis during the AV condition (*p* < 0.001, uncorrected) within appropriate anatomical atlas using Anatomical Automatic Labeling (AAL)^64^ for Heschl and Calcarine, and Anatomy Toolbox^65^ for MT. The 6 mm-radius spheres were created around these centre coordinates, and defined as ROIs. To define the left STS ROI, we adopted the mean criterion, which is when the BOLD signals for multisensory stimulation exceeds the mean of both unisensory responses (AV > mean (AO + visual-only (VO)))^66^ because a previous study^66^ showed that the mean criterion was suitable for revealing STS multisensory integration site. First, we performed conjunction analysis of the AO condition and the VO condition (*p* < 0.001, uncorrected, conjunction null) using a factorial design matrix in each group. Then, contrast of AV > mean (AO + VO) was calculated in the conjunction area using a liberal threshold (*p* < 0.05, uncorrected) because we had already set the threshold at *p* < 0.001 for conjunction analysis. The peak within 6 mm of the mean group maxima in STS was set individually due to large individual differences of STS location^55^. Then, a 6 mm-radius sphere located around this point was defined as the participant’s STS ROI. Calcarine and Heschl were defined in each group based on group analyses. MT was also defined based on group analysis, but using group comparison (Japanese speaker – English speaker under AV condition). The time courses of these ROIs were extracted after regressing out the WM, CSF, effects of condition, and movement parameters. Correlation coefficients between two ROIs were z-transformed, with one- and two-sample *t*-tests examining the within- and between-group differences in connectivity. Significant connectivity was defined using a threshold of *p* < 0.05 (two-tailed), and were corrected for multiple comparisons using the seed-level false discovery rate (FDR) method.

## Additional Information

**How to cite this article**: Shinozaki, J. *et al*. Impact of language on functional connectivity for audiovisual speech integration. *Sci. Rep.* **6**, 31388; doi: 10.1038/srep31388 (2016).

## Supplementary Material

Supplementary Information

## Figures and Tables

**Table 1:** Brain areas activated under the AV, AO, and VO conditions.

Cluster Size	Locations (Brodmann area)	*x*	*y*	*z*	*Z*-Value
Brain areas activated by AV stimuli in native English speakers*					
5551	L. Cuneus (18)	−6	−102	−6	6.58
	L. Cuneus (17)	−9	−99	−9	6.54
	R. Lingual Gyrus (18)	3	−84	−9	6.27
	R. Middle Temporal Gyrus (21)	57	−18	−3	6.06
	R. Inferior Occipital Gyrus (19)	36	−81	−9	5.74
	L. Middle Temporal Gyrus (22)	−54	−33	6	5.50
	R. Putamen	27	−9	−6	5.45
	R. Superior Temporal Gyrus (41)	51	−33	9	5.41
	R. Superior Temporal Gyrus (22)	66	−36	6	5.25
	L. Superior Temporal Gyrus (22)	−66	−39	6	5.13
450	R. Precentral Gyrus (6)	45	−21	66	5.62
209	L. Medial Frontal Gyrus (6)	−6	3	57	4.87
	R. Medial Frontal Gyrus (6)	9	3	54	4.16
64	L. Cerebellum	−12	−63	−45	4.53
Brain areas activated by AV stimuli in native Japanese speakers*					
3664	L. Cuneus (18)	−15	−102	3	7.10
	R. Cuneus (17)	12	−99	0	6.31
	L. Inferior Occipital Gyrus (17)	−12	−90	−15	5.90
	R. Fusiform Gyrus (37)	42	−48	−18	5.45
	R. Middle Occipital Gyrus (37)	39	−69	−3	5.28
	L. Superior Temporal Gyrus (13)	−51	−42	15	5.10
	L. Fusiform Gyrus (37)	−45	−57	−24	5.07
	L. Superior Temporal Gyrus (22)	−60	−42	9	4.98
	L. Superior Temporal Gyrus (41)	−42	−36	9	4.97
1241	R. Superior Temporal Gyrus (22)	66	−36	15	6.06
	R. Superior Temporal Gyrus (41)	54	−30	12	5.99
398	R. Precentral Gyrus (4)	33	−27	57	5.97
289	R. Medial Frontal Gyrus (6)	9	−6	60	5.06
	L. Medial Frontal Gyrus (6)	−12	0	57	4.50
Brain areas showing greater activation in native English speakers than in native Japanese speakers under the AV condition**					
	No suprathreshold voxels				
Brain areas showing greater activation in native Japanese speakers than in native English speakers under the AV condition**					
29	L. Inferior Temporal Gyrus (37)	−51	−69	−3	3.40
Brain areas activated by AO stimuli in native English speakers*					
1736	L. Cuneus (17)	−9	−99	−6	6.64
	R. Cuneus (18)	12	−102	6	6.39
	L. Cuneus (18)	−12	−105	6	6.38
	R. Lingual Gyrus (17)	6	−93	−9	6.10
	L. Fusiform Gyrus (37)	−39	−51	−24	4.18
706	R. Middle Temporal Gyrus (21)	57	−30	0	5.79
	R. Superior Temporal Gyrus (22)	66	−36	6	5.45
564	L. Superior Temporal Gyrus (22)	−57	−27	6	5.40
	L. Superior Temporal Gyrus (41)	−39	−36	3	3.99
368	R. Precentral Gyrus (4)	42	−21	69	5.24
216	L. Superior Frontal Gyrus (6)	−6	6	57	4.82
	R. Medial Frontal Gyrus (6)	9	−9	54	3.15
76	R. Putamen	24	−3	12	4.41
72	R. Cerebellum	42	−51	−33	4.15
Brain areas activated by AO stimuli in native Japanese speakers*					
1366	R. Lingual Gyrus (18)	9	−87	−9	5.93
	L. Cuneus (18)	−15	−102	3	5.48
	L. Fusiform Gyrus (37)	−42	−54	−21	4.42
563	R. Superior Temporal Gyrus (22)	63	−33	9	5.07
	R. Superior Temporal Gyrus (41)	57	−21	3	4.90
349	R. Precentral Gyrus (4)	30	−27	66	5.06
295	L. Medial Frontal Gyrus (32)	−6	6	51	4.81
	R. Medial Frontal Gyrus (6)	6	−6	60	4.53
	L. Medial Frontal Gyrus (6)	−6	−9	66	4.02
336	L. Superior Temporal Gyrus (22)	−57	−45	9	4.53
	L. Superior Temporal Gyrus (42)	−60	−30	6	4.02
185	R. Fusiform Gyrus (37)	42	−48	−18	4.46
Brain areas showing greater activation in native English speakers than in native Japanese speakers under the AO condition**					
27	L. Anterior Cingulate (24)	−6	36	9	3.76
Brain areas showing greater activation in native Japanese speakers than in native English speakers under the AO condition**					
10	R. Middle Occipital Gyrus (19)	36	−87	6	3.57
Brain areas activated by VO stimuli in native English speakers*					
2368	L. Cuneus (17)	−9	−99	−9	6.50
	R. Lingual Gyrus (18)	15	−87	−12	6.39
	L. Cuneus (18)	−9	−105	3	6.34
	R. Lingual Gyrus (17)	9	−96	−9	5.94
	R. Fusiform Gyrus (37)	42	−51	−18	5.05
	L. Fusiform Gyrus (37)	−42	−54	−24	4.85
	R. Middle Temporal Gyrus (19)	51	−69	6	4.75
470	R. Precentral Gyrus (6)	36	−18	72	5.71
	R. Precentral Gyrus (4)	42	−24	66	5.60
291	R. Superior Frontal Gyrus (6)	3	6	60	5.25
196	R. Middle Temporal Gyrus (22)	54	−39	6	4.71
65	L. Caudate	−6	6	18	4.66
83	L. Precentral Gyrus (6)	−54	−3	48	4.55
84	R. Putamen	30	0	−3	4.38
51	L. Caudate	−18	−27	27	4.03
Brain areas activated by VO stimuli in native Japanese speakers*					
3681	R. Lingual Gyrus (18)	6	−87	−9	6.45
	L. Cuneus (18)	−15	−102	3	6.28
	R. Fusiform Gyrus (37)	42	−48	−18	6.19
	L. Middle Occipital Gyrus (37)	−51	−72	0	6.07
	L. Fusiform Gyrus (37)	−42	−51	−21	5.63
	R. Middle Occipital Gyrus (37)	54	−69	0	4.90
	R. Superior Temporal Gyrus (22)	54	−39	9	4.49
575	L. Medial Frontal Gyrus (32)	−6	6	51	6.06
	R. Medial Frontal Gyrus (6)	6	−6	60	5.56
	L. Superior Frontal Gyrus (6)	−6	−6	66	4.97
908	L. Precentral Gyrus (6)	−54	−3	48	5.67
1210	R. Precentral Gyrus (4)	30	−27	66	5.23
116	L. Putamen	−21	−3	18	5.05
351	L. Superior Parietal Lobule	−33	−57	48	4.94
94	L. Cerebellum	−21	−60	−51	4.89
154	R. Inferior Parietal Lobule	33	−54	45	4.73
68	R. Cerebellum	21	−69	−48	4.40
Brain areas showing greater activation in native English speakers than in native Japanese speakers under the VO condition**					
86	L. Medial Frontal Gyrus (10)	−12	42	12	3.99
Brain areas showing greater activation in Japanese native speakers than in native English speakers under the VO condition**					
127	L. Inferior Temporal Gyrus (37)	−54	−69	−3	4.38
85	R. Middle Temporal Gyrus (37)	54	−57	3	4.15
76	L. Superior Parietal Lobule (7)	−30	−57	54	4.01
46	R. Superior Occipital Gyrus (19)	39	−81	24	3.93
75	R. Middle Frontal Gyrus (8)	51	9	45	3.89
24	R. Superior Parietal Lobule (7)	33	−57	63	3.80
16	L. Inferior Frontal Gyrus (46)	−48	30	12	3.63
10	L. Cuneus (17)	−24	−84	9	3.54
11	L. Cerebellum	−24	−69	−48	3.35

*Significant at *p* < 0.05 (cluster-level, family-wise error corrected for multiple comparisons) at *p* < 0.001 (voxel-level, uncorrected). **Significant at *p* < 0.001 (voxel-level, uncorrected). Cluster size = number of voxels; *x*, *y*, *z* = MNI coordinates.
