# Supplementary material for: Impact of language on functional connectivity for audiovisual speech integration
Source: Sci Rep. 2016 Aug 11;6:31388. doi: 10.1038/srep31388 (PMC4980767; doi:10.1038/srep31388)
Supplement: Supplementary Information [file srep31388-s1.pdf]

# Supplementary Information

## Impact of language on functional connectivity for audiovisual speech integration

Jun Shinozaki, Nobuo Hiroe, Masa-aki Sato, Takashi Nagamine, and Kaoru Sekiyama

## Analysis of reaction times using a 3-way ANOVA

As participants were given essentially the same instructions for the AV and AO conditions (i.e., to respond as soon as possible after listening to the auditory syllable), the RTs of the AV and AO conditions were compared. We conducted a 3-way ANOVA, including the within-subject factors CONDITION (AV, AO), TALKER (native, non-native), and the between-subject factor GROUP (English speaker, Japanese speaker) for RTs. There was no significant effect of TALKER ($F_{1, 40}$ = 2.589, $p$ = 0.115, partial $\eta^2$ = 0.061). However, when we divided TALKER into English talker, called Mik, and Japanese talker, called Ter, a 3-way ANOVA revealed a significant effect of TALKER ($F_{1, 40}$ = 13.115, $p$ = 0.001, partial $\eta^2$ = 0.247), as well as CONDITION ($F_{1, 40}$ = 5.000, $p$ = 0.031, partial $\eta^2$ = 0.111) and GROUP ($F_{1, 40}$ = 4.767, $p$ = 0.035, partial $\eta^2$ = 0.106), indicating individual differences in talkers (i.e., one talker's speech was more quickly perceived than the other's) were significant, while the effect of whether a talker was native or non-native was not significant. As the interaction between GROUP and TALKER (individual difference of talker) was not significant ($F_{1, 40}$ = 2.589, $p$ = 0.115, partial $\eta^2$ = 0.061), we pooled the TALKER data in for this analysis. We could decrease the effect of individual difference of talker by pooling TALKER.

## Comparisons of Caucasian and Asian in the native English speakers

The native English speakers (n = 20 in behavioural experiment) consisted of Caucasians and Asians (n = 5). We tested the subgroup differences in the task performance data between Caucasian English-speakers and Asian English-speakers using two sample *t*-test. We used parametric *t*-test because the data showed homogeneity of variance as indicated by Levene's test and normal distribution as indicated by Kolmogorov-Smirnov test and Shapiro-Wilk test.

There was no significant subgroup difference in the reaction time for AV ($t_{18} = 1.588$, $p = 0.130$, Cohen's $d = 0.82$), AO ($t_{18} = 1.029$, $p = 0.317$, $d = 0.53$), and VO ($t_{18} = 1.940$, $p = 0.068$, $d = 1.00$); however, large sized effects were found. Therefore, these results suggested that there might be significant subgroup differences in replication studies using larger sample size.

## Comparisons of behavioural data between inside and outside the scanner

The behavioural analysis was intended for subjects who attended both behavioural experiment (outside the scanner) and fMRI experiment (inside the scanner) (19 English speakers and 18 Japanese speakers) (Fig. S1). We conducted ANOVA with the within-group factor EXPERIMENT (inside or outside the scanner) and the between-group factor GROUP (English speakers or Japanese speakers) in each condition. In the AV condition, neither EXPERIMENT ($F_{1, 35} = 2.743$, $p = 0.107$, partial $\eta^2 = 0.073$) nor the EXPERIMENT × GROUP interaction ($F_{1, 35} = 0.262$, $p = 0.612$, partial $\eta^2 = 0.007$) was significant, but English speakers responded significantly faster than Japanese speakers (F1, 35 = 4.238, p = 0.047, partial $\eta^2 = 0.108$). Similarly, in the AO condition, neither EXPERIMENT ($F_{1, 35} = 2.684$, $p = 0.110$, partial $\eta^2 = 0.071$) nor the EXPERIMENT × GROUP interaction ($F_{1, 35} = 0.010$, $p = 0.923$, partial $\eta^2 < 0.001$) was significant, but English speakers responded significantly faster than Japanese speakers ($F_{1, 35} = 4.807$, $p = 0.035$, partial $\eta^2 = 0.121$). In the VO condition, neither EXPERIMENT ($F_{1, 35} = 3.647$, $p = 0.064$, partial $\eta^2 = 0.094$) nor the EXPERIMENT × GROUP interaction ($F_{1, 35} = 0.083$, $p = 0.775$, partial $\eta^2 = 0.002$) was significant, but English speakers responded significantly faster than Japanese speakers ($F_{1, 35} = 8.012$, $p = 0.008$, partial $\eta^2 = 0.186$). These findings indicate that RTs were not significantly different between the behavioural experiment and the fMRI experiment. We did not conduct any statistical analysis of accuracy because there was a ceiling effect due to the simplicity of the task.

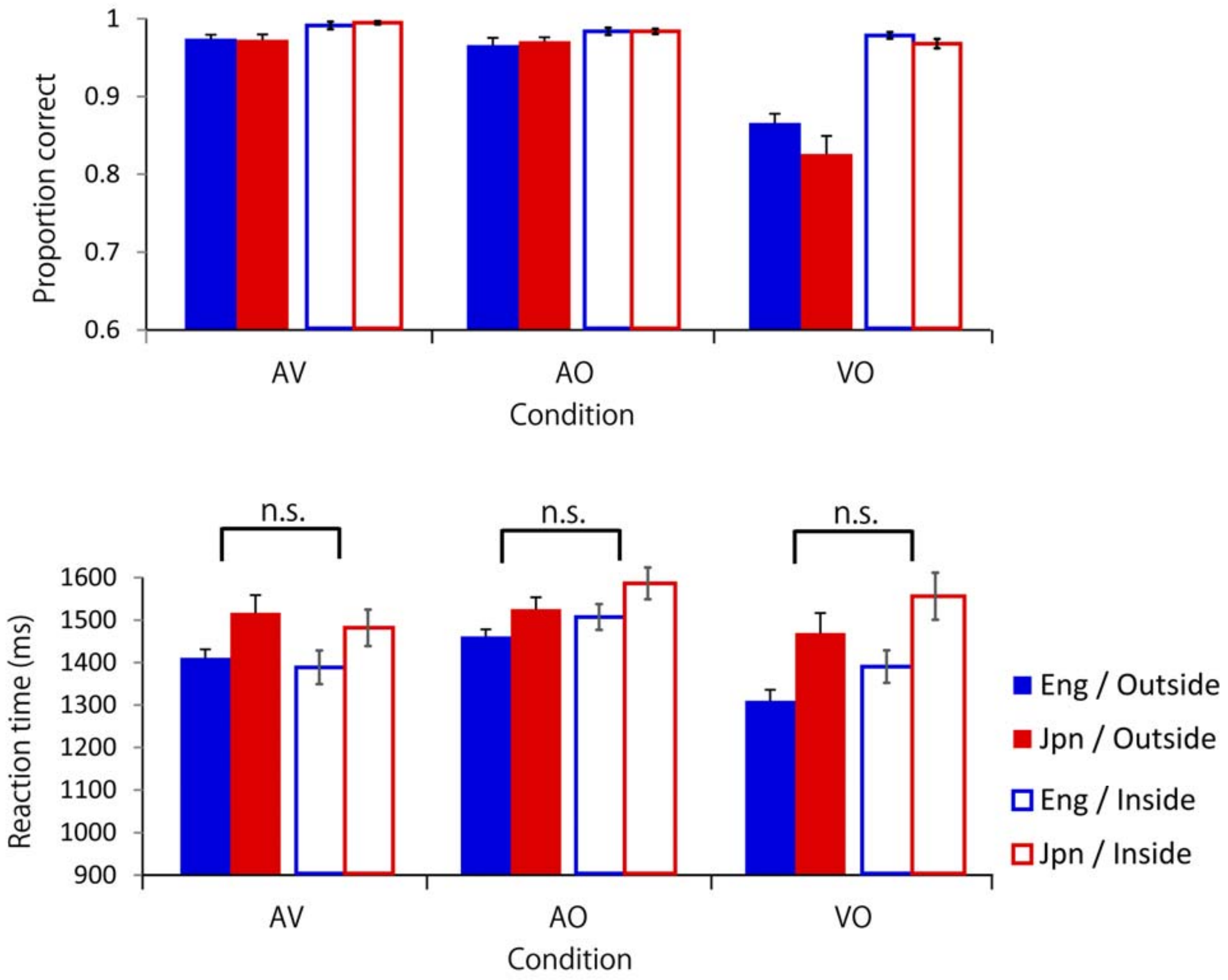


Fig. S1: Proportion correct and reaction times inside/outside scanner. Error bars represent standard error. n.s.; not significant. Eng: English speakers. Jpn; Japanese speakers.

## BOLD responses in the ROIs

STS was defined individually based on the group analysis of each group using the mean criterion. Calcarine, Heschl, and Thalamus were defined based on the group analysis of each group in the AV condition. MT was defined by subtraction of English speakers from Japanese speakers in the AV condition.

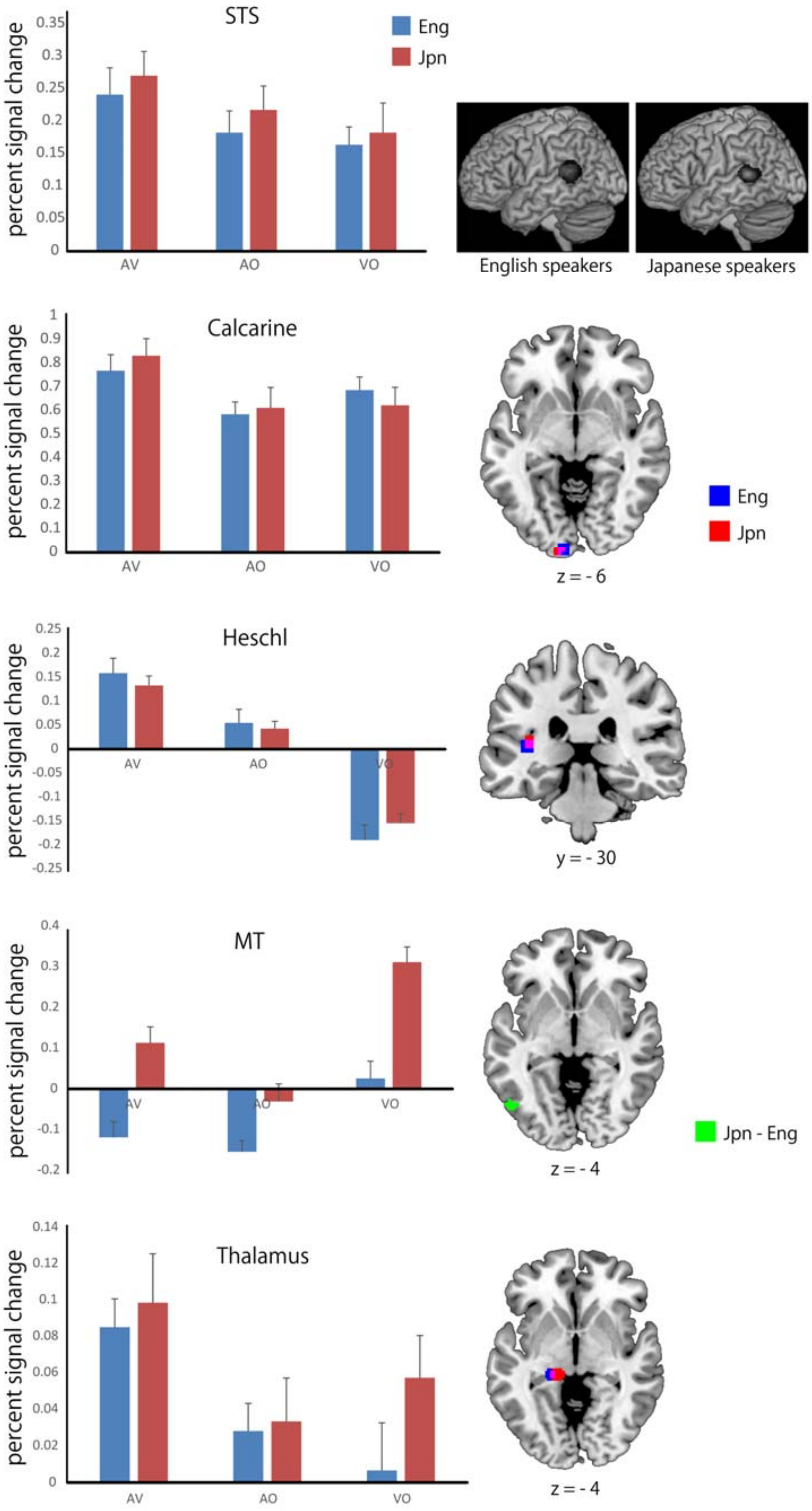


Fig. S2: BOLD responses in the ROIs: The percent signal changes in each condition in each ROI are shown. Eng: English speakers. Jpn: Japanese speakers.

## Additional analysis of functional connectivity including left thalamus

To investigate the involvement of the thalamus in functional connectivity between auditory and visual areas, we defined Thalamus ROI using the same method as in the main manuscript (see Methods). First, we defined peak coordinate of activity in group analysis under AV condition ($p < 0.001$, uncorrected) within anatomical atlas (left thalamus in Automated Anatomical Labeling (AAL)[1]) in each group. Then, we created a 6-mm radius sphere centred on this peak, and defined as Thalamus ROI.

Figure S3 shows functional connectivity map including left STS, Calcarine, MT, Heschl, and Thalamus under the AV condition. In English speakers, significant Thalamus-Calcarine ($p = 0.012$, $Z = 0.10$ ($Z$: Fisher's $Z$-transformation of correlation coefficients $r$)), Thalamus-MT ($p = 0.044$, $Z = 0.09$), and Thalamus-Heschl ($p = 0.013$, $Z = 0.08$) connectivities were found (seed-level FDR corrected (two-tailed)), while in Japanese speakers, none of these connectivities were found (Thalamus-Calcarine ($p = 0.509$, $Z = 0.03$), Thalamus-MT ($p = 0.770$, $Z = –0.01$), and Thalamus-Heschl ($p = 0.150$, $Z = 0.08$)).

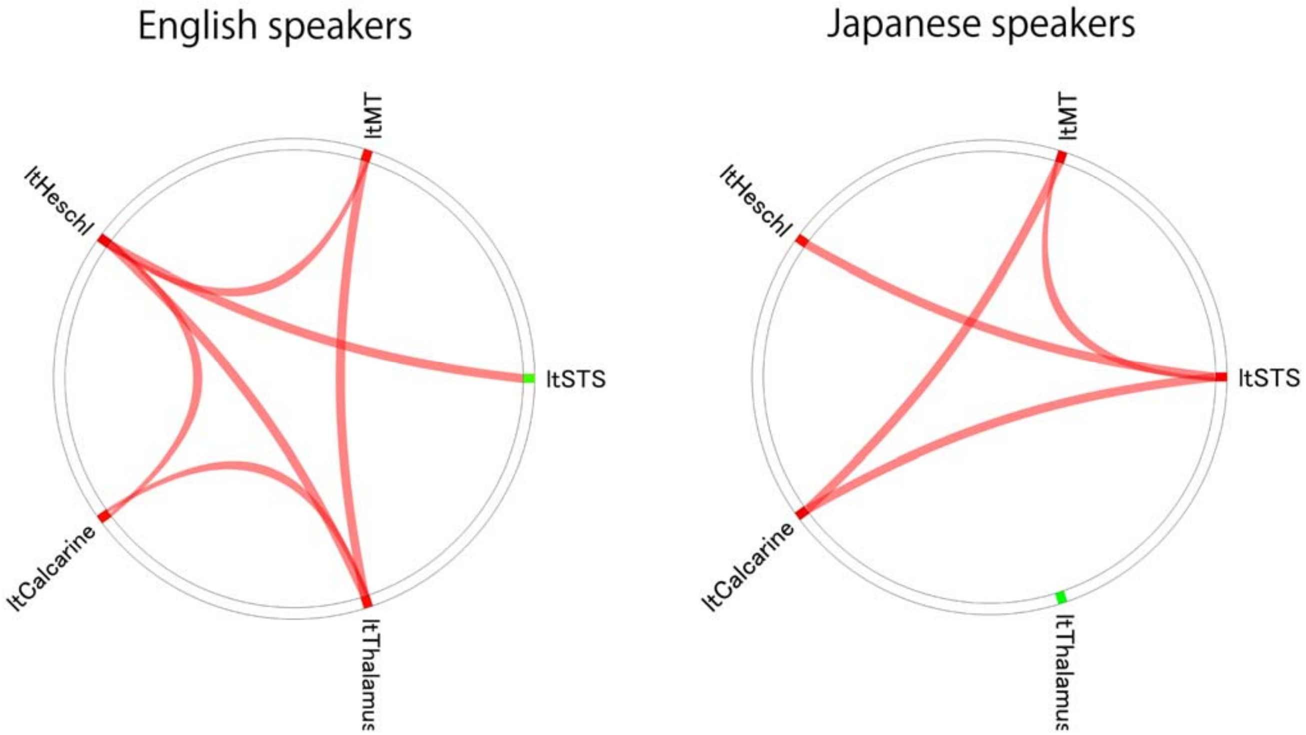


Fig. S3: Functional connectivity. All connectivities shown in red mean a positive correlation. Consistent with the analysis of group differences in functional connectivity without Thalamus (see

main manuscript), only the MT-Heschl connectivity was also significantly stronger in English speakers than Japanese speakers in this analysis including Thalamus ($p < 0.001$, $Z = 0.21$).

## Reference

1. Tzourio-Mazoyer, N. et al. Automated anatomical labeling of activations in SPM using a macroscopic anatomical parcellation of the MNI MRI single-subject brain. NeuroImage 15, 273-289, doi:10.1006/nimg.2001.0978 (2002).
